# Carbon dioxide contrast medium for endovascular treatment of ilio-femoral occlusive disease

**DOI:** 10.6061/clinics/2015(10)03

**Published:** 2015-10

**Authors:** Cynthia de Almeida Mendes, Alexandre de Arruda Martins, Marcelo Passos Teivelis, Sergio Kuzniec, Andrea Yasbek Monteiro Varella, Alexandre Fioranelli, Nelson Wolosker

**Affiliations:** I Hospital Israelita Albert Einstein, Cirurgia Vascular, São Paulo/, SP, Brazil; II Hospital Municipal Dr Moyses Deutsch, Serviço de Cirurgia, São Paulo/, SP, Brazil; III Hospital das Clinicas da Faculdade de Medicina da Universidade de São Paulo, Serviço de Cirurgia Vascular, São Paulo/, SP, Brazil

**Keywords:** Contrast Media, Carbon Dioxide, Iliac, Endovascular

## Abstract

**OBJECTIVES:**

Compare the use of carbon dioxide contrast medium with iodine contrast medium for the endovascular treatment of ilio-femoral occlusive disease in patients without contraindications to iodine.

**MATERIALS AND METHODS:**

From August 2012 to August 2014, 21 consecutive patients with ilio-femoral occlusive disease who were eligible for endovascular treatment and lacked contraindications to either iodine contrast or carbon dioxide were randomized into the carbon dioxide or iodine groups and subjected to ilio-femoral angioplasty.

We analyzed the feasibility of the procedures, the surgical and clinical outcomes, the procedure lengths, the endovascular material costs, the contrast costs and the quality of the angiographic images in each group.

**RESULTS:**

No conversions to open surgery and no contrast media related complications were noted in either group. A post-operative femoral pulse was present in 88.9% of the iodine group and 80% of the carbon dioxide group. No differences in procedure length, endovascular material cost or renal function variation were noted between the groups. Four patients in the carbon dioxide group required iodine supplementation to complete the procedure. Contrast media expenses were reduced in the carbon dioxide group. Regarding angiographic image quality, 82% of the carbon dioxide images were graded as either good or fair by observers.

**CONCLUSIONS:**

The use of carbon dioxide contrast medium is a good option for ilio-femoral angioplasty in patients without contraindications to iodine and is not characterized by differences in endovascular material costs, procedure duration and surgical outcomes. In addition, carbon dioxide has lower contrast expenses compared with iodine.

## INTRODUCTION

Arteriosclerotic occlusive disease of the ilio-femoral arteries is a common cause of ischemic symptoms in the lower limbs. Revascularization is the treatment of choice for patients when either limiting intermittent claudication unresponsive to clinical treatment or critical limb ischemia is present 1-4.

Iodine contrast medium (ICM) is considered the gold standard for endovascular treatments worldwide. An alternative that has been used within the last 15 years is carbon dioxide (CO_2_), which is used for patients with renal impairment or hypersensitivity to iodine because it is non-nephrotoxic and non-allergenic 5. With improvements in both imaging equipment and injection techniques, CO_2_ has even been used in patients without contraindications to ICM for the detection of endoleaks 6, femoral-popliteal angioplasty 7,8, cavography for vena cava filter placement 9 and investigation of the biliary tree 10.

To date, no randomized prospective studies have compared the outcomes of CO_2_ contrast media and ICM use for endovascular revascularization of the ilio-femoral segment in patients with no contraindications to ICM. Therefore, to determine whether CO_2_ is as effective as iodine contrast media for ilio-femoral angioplasties, we performed a randomized prospective study involving patients with ilio-femoral occlusive disease who were eligible for either type of contrast (ICM or CO_2_). We analyzed the feasibility of the procedures, the surgical and clinical outcomes, the procedure lengths, the endovascular material costs, the contrast costs and the quality of the angiographic images.

## MATERIAL AND METHODS

From August 2012 to August 2014, 21 consecutive patients with ilio-femoral disease (all with atherosclerotic disease) with arterial lesions classified as Trans-Atlantic Inter-Society Consensus Document on Management of Peripheral Arterial Disease (TASC) A, B, C or D (identified via preoperative angiotomography) and adequate runoff underwent endovascular ilio-femoral revascularization in a prospective randomized controlled study. This study was approved by the Ethics Committee for Analysis of Research Projects on Human Experimentation of our institution (Clinical Trials Identifier: NCT01861327). None of these patients exhibited severe chronic obstructive pulmonary disease, kidney failure, heart failure, or pregnancy

The patients were randomized into the following two groups: a CO_2_ group and an ICM group, according to the contrast medium selected for the procedure. All patients provided informed consent and agreed to the use of either contrast medium. Randomization was performed using a computer-generated list immediately prior to the beginning of the surgery. Eighteen of the 21 patients had no palpable femoral pulses in the limb that was revascularized. Two patients in the CO_2_ group and one patient in the ICM group had weak palpable femoral pulses pre-operatively. Table 1 presents the demographic, clinical and laboratory profiles of the patients in our study sample who were subjected to either ICM or CO_2_.

All procedures were performed in an endovascular suite operating room utilizing a Philips Allura Xper FD system (Philips Healthcare, Best, the Netherlands) under general anesthesia with cardiac monitoring, invasive arterial pressure monitoring and bladder catheterization.

The patients underwent ilio-femoral angioplasties by the same surgical team (four surgeons) using the same surgical technique throughout the study in the same hospital (Hospital Israelita Albert Einstein, São Paulo, Brazil). An ipsilateral puncture of the common femoral artery was used for access in 19 of the 21 patients. In one case, an ipsilateral puncture was unsuccessful; therefore, we opted for contralateral femoral access. In another case, a contralateral puncture was the initial choice due to the presence of an atherosclerotic lesion in the external iliac artery.

Standard surgical technique was used in all cases. After an ultrasound guided arterial puncture and the insertion of a 6-French introducer access sheath, systemic heparinization was performed followed by initial angiography with the appropriate contrast medium. The lesion was crossed using catheters and hydrophilic guide wires followed by the selective delivery of a self-expanding stenting and pre- and post-dilatation with an angioplasty balloon. At the end of the procedure, the heparinization was reversed, and local manual compression was performed for 30 minutes after removal of the introducer. All of the endovascular materials were provided by Cook Medical, Inc.®.

The CO_2_ injection was performed manually without a specific pump system. A cylinder with medicinal CO_2_, a particle filter (Millex® Durapore® hydrophilic 0.22 μm pore) and a three-way stopcock were used for CO_2_ aspiration, and the entire procedure was performed inside a bowl with saline solution. After capturing the required volume of CO_2_, an additional 3 to 5 ml of saline solution was aspirated into the syringe to provide a water seal as the tip was held down. This process allowed for the creation of a physical barrier between the room air and the carbon dioxide, which was independent of the manual compression and safe from air contamination 11. Twenty-milliliter syringes were used for the CO2 intra-arterial contrast injection, which was performed using either a femoral introducer or a catheter.

The injection of ICM was performed manually using 10-ml syringes with 3 ml of iodinated contrast media and 7 ml of saline solution per injection. The ICM used in all cases was Omnipaque 300 (Iohexol), a nonionic low osmolar contrast agent routinely used by our service.

Immediately after the operation, all patients were referred to the intensive care unit for at least 24 hours in accordance with our institutional research protocol for vascular surgery and received intravenous fluids following a fixed protocol for renal protection, regardless of the contrast medium used. The protocol for renal protection included intravenous fluids pre-procedure with endovenous acetylcysteine and intravenous fluid maintenance for 24 hours. Thereafter, the patient remained hospitalized for the time needed, with daily renal function, blood count and electrolyte monitoring for at least 72 hours.

The endovascular material used in each intervention and the volumes of ICM or CO_2_ were precisely recorded for further analysis.

The endovascular material costs included puncture needles, sheaths, angioplasty balloons, catheters, insufflating syringes and stents used during the endovascular procedure. The contrast costs included the total cost of the contrast used during the procedure. It is important to emphasize that because ICM is required for balloon filling during angioplasties, we included the price of one 20-ml flask of ICM for each patient in the CO_2_ group (irrespective of ICM supplementation).

Procedure length was measured based on the amount of time elapsed from the arterial puncture until the end of manual compression.

We analyzed creatinine clearance levels between the groups during the pre- and post-operative periods as well as creatinine clearance variations between the post- and pre-operative periods (delta clearance).

All procedures were recorded on DVD and the recorded angiography procedures (of both groups) were analyzed by two observers who did not participate in the intervention and had no experience with the use of CO_2_ contrast. The observers assigned a score ranging from 1 to 3 to each image (scoring was only based on the iliac-femoral segment; therefore, 11 CO2 images were scored, i.e., one image per patient). A score of 1 was considered *poor* and was assigned when there was significant loss of definition within the vessels or collateral circulation, which precluded performing the procedure. A score of 2 was considered *fair* and was assigned when there was some loss of definition within the vessels or collateral circulation, which did not preclude the intervention. A score of 3 was considered *good* and was assigned when there was good contrast within the vessels or collateral circulation.

We evaluated the following clinical outcomes in both groups: the feasibility of the procedures, the complications, the surgical outcomes (femoral pulses and ankle brachial index (ABI)), the pre- and post-operative creatinine clearance, the contrast volume, the quality of the angiographic images, the procedure costs and the contrast media costs.

### Statistical Analysis

Categorical variables were expressed as absolute frequencies and percentages and compared between groups via Fisher's exact test. Numerical variables were expressed as medians and interquartile ranges and compared via the nonparametric Mann-Whitney U test. The level of significance was 5%.

## RESULTS

Endovascular treatment was performed in 21 patients (11 patients in the CO_2_ group and 10 patients in the ICM group) with ilio-femoral disease. The percentages of overall technical success in the CO_2_ and ICM groups were 90.9% and 90%, respectively, with only one procedure failure per group. These failures were the only patients who did not receive a self-expanding stent. No procedure-related deaths were noted and none of the patients presented with major clinical or surgical complications. Conversion to open surgery was not necessary in any of the cases and no contrast media-related complications were noted in either group (CO_2_ or ICM).

The surgical outcomes were satisfactory in both groups. The presence of palpable femoral pulses measured immediately following each procedure indicated that the technical success rate was similar for both groups, as presented in Table 2. (The three patients with weak femoral pulses pre operatively presented with strong palpable femoral pulses post operatively and were considered technical successes.) Variability (difference between post-operative and pre-operative ABI) was higher for the ICM group (0.40 *vs*. 0.16, *p*=0.044).

For the ICM group, the median iodine contrast volume was 34.25 ml (range: 9 to 69 ml); for the CO_2_ group, the median CO2 volume was 60 ml (range: 23 to 130 ml). In four (36.4%) CO_2_ patients, the use of iodine contrast was necessary to complete the procedure; however, none of these patients required more than 15 ml of iodine (range: 3 to 15 ml). The median iodine contrast volume used for the CO_2_ patients who required iodine supplementation was 6.5 ml.

Creatinine clearance during the postoperative period did not differ significantly between the groups and remained stable during the post-operative period in both groups, as presented in Table 3.

The delta clearance (creatinine clearance after the procedure minus clearance before the procedure) was positive for the CO_2_ group (2.01) and zero for the ICM group; however, this difference was not statistically significant (*p*=0.44).

Procedure length did not differ significantly between the groups (median length was 75 minutes for the CO_2_ group and 70 minutes for the ICM group, *p*=0.92), indicating that CO_2_ does not increase procedure duration. We also analyzed the procedure lengths for the different TASC classifications in each group and observed no significant differences.

The costs of the endovascular materials were similar for both groups, indicating that CO_2_ does not increase procedure costs. However, the contrast medium costs were significantly increased for the ICM group compared with the CO_2_ group (see Table 4).

**Figure 1:**
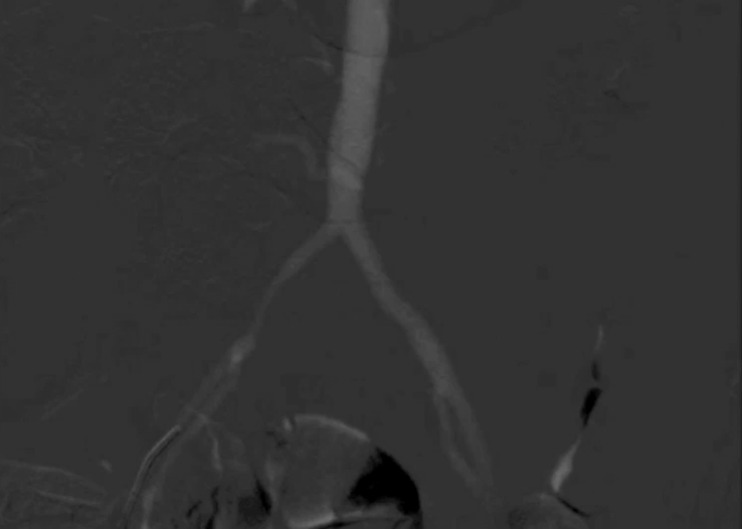
Carbon dioxide angiography.

Regarding angiographic image quality, which is presented in Table 5, 82% of the CO_2_ angiography images were graded as either good or fair by both observers, with high inter-observer image quality concordance. All iodine arteriograms were graded as good by both observers. Figure 1 depicts an example of CO_2_ angiography.

## DISCUSSION

Treating patients with renal impairment or iodine related hypersensitivity remains a challenge when performing both diagnostic and therapeutic endovascular procedures. The use of CO_2_ as an alternative contrast medium to ICM has provided a solution for these situations. Given that CO_2_ is a good option for these patients, we aimed to investigate the possibility of the more widespread use of CO_2_ by treating patients with no contraindications to either CO_2_ or ICM.

In this study, we observed that the use of CO_2_ did not change surgical outcomes given that the patients in the CO_2_ group exhibited no differences in post-operative femoral pulses compared with the patients who received iodine contrast. The variation in ABI between the groups may be explained by differences in distal outflow (i.e., patients presented with different patterns of femoro-popliteal and infra-genicular stenosis/occlusion). In addition, procedure length was not extended in the CO_2_ group. Pain during the injection of CO_2_ was not assessed because the patients were under general anesthesia. However, some of the patients could have received local anesthesia (and this assessment could have been undertaken). Due to the institutional research protocol (which mandated general anesthesia and an overnight stay on ICU for every vascular surgery patient), we were unable to compare discomfort (e.g., pain) during the procedure between the groups.

No significant differences were noted between the patients treated with CO_2_ or iodine with respect to renal function during the post-operative period. In the ICM group, we did not observe any changes. The lack of alteration was most likely because of either a small sample size or the administration of a low volume of iodine contrast, as numerous studies have demonstrated the nephrotoxicity of ICM.

Endovascular material costs were similar for both groups, demonstrating that the use of CO_2_ does not increase procedure costs. However, the contrast media costs for the ICM group were significantly higher compared with the CO_2_ group, even after including the price of one 20-ml flask of ICM for balloon filling for each patient in the CO_2_ group.

The evaluation of the angiographic images by the observers indicated that most of the images in the CO_2_ group (82%) were evaluated as either good or fair; therefore, it may be possible to perform the procedure with only CO_2_ in these cases. It is noteworthy that 63% of images for observer 1 and 45% for observer 2 were considered good with CO_2_ and that all images with iodine were rated as good. However, our aim was to assess the feasibility of C0_2_ not its superiority to iodine contrast media.

Images were considered poor with CO_2_ in patients with long occlusions (2 patients with TASC D lesions for whom the filling of the distal vessels with CO_2_ was difficult to assess). Additional poor images were observed at the beginning of our series, at which time we supplemented patients with iodine to verify vessel patency. Although the learning curve of this procedure may be overcome with experience, it is possible that CO_2_ may not replace iodine in TASC D lesions. This limitation might be a problem for allergic patients but may also benefit patients with borderline renal function for whom iodine contrast media usage may be significantly reduced.

The CO_2_ contrast agent is a gas; therefore, its manipulation requires the use of an unusual technique. Most vascular surgeons are not accustomed to working with a gaseous contrast medium, as its manipulation requires care to prevent air contamination 12. In addition, most surgeons lack the training needed to obtain good quality images.

Some CO_2_ injection pumps have been developed 13-15, but they are expensive and difficult to use. We have developed and published a simple homemade delivery system for CO_2_ in endovascular procedures, which uses materials that may be found in any hospital and are both cheap and reproducible. We believe that systems such as ours may lead to more widespread use of CO_2_ as a contrast agent in endovascular procedures.

When performed by experienced physicians familiar with its use, CO_2_ may provide angiographic image quality comparable to that achieved using iodinated contrast media.

We consider CO_2_ as effective as ICM within the femoro-popliteal territory. We demonstrated that CO_2_ is a viable contrast option for ilio-femoral lesion treatment and observed outcomes similar to those noted with iodine contrast. Complications following the use of intra-arterial CO_2_ have been reported, including *vapor-lock*, which occurs upon entrapment of CO_2_ at the origin of a vessel, thereby impairing flow and potentially resulting in ischemia. This condition may be mild and cause only transient pain or may present as significant bowel ischemia 16,17. However, no adverse events were observed in our series secondary to the use of this particular contrast media. We routinely waited 3 minutes between CO_2_ injections to avoid *vapor-lock* and any subsequent complications.

We believe that vascular surgeons should be trained during their residency in vascular surgery to acquire the technical ability to use CO_2_. The technique is unusual, but its learning curve is not steep. The technique may be useful for vascular surgeons searching for the best contrast option for each patient, as opposed to being limited to the use of only one type of contrast.

Given this study's small sample size and the absence of a longer follow up period with respect to renal function, additional studies supporting our findings are encouraged.

Our study demonstrated that CO_2_ is a good alternative to ICM in the endovascular treatment of ilio-femoral occlusive disease (except in TASC D patients, for whom iodine was often but not always necessary to complete the procedure). CO_2_ is characterized by similar endovascular material costs, procedure lengths and surgical outcomes as well as and reduced contrast medium costs compared with iodine contrast.

## Figures and Tables

**Table 1:** Demographic and clinical profiles.

	GROUPS		
	CO_2_ (n=11)	ICM (n=10)	*p*-value
Age average (IIQ)	48 (43.5-60.0)	69 (60.0-75.0)	0.005 *
Age range (years)	37-73	58-79	
Median BMI (kg/m^2^)	21.72	24.17	0.26 *
Diabetes (%)	6 (54.5)	4 (40.0)	0.67 #
Hypertension (%)	7 (63.6)	8 (80.0)	0.63 #
Dyslipidemia (%)	4 (36.4)	4 (40.0)	>0.99 #
Coronary disease (%)	0 (0.0)	1 (10.0)	0.48 #
History of tobacco use (%)	10 (90.9)	7 (70.0)	0.42 #
Intermittent claudication (%)	3 (27.3)	3 (30.0)	>0.99 #
Critical ischemia	8(72.7)	7(70.0)	0,63 #
TASC A lesion (%)	6 (54.5)	2 (20.0)	0.24 #
TASC B lesion (%)	2 (18.2)	2 (20.0)	0.24 #
TASC C lesion (%)	0 (0.0)	3 (30.0)	0.24 #
TASC D lesion (%)	3 (27.3)	3 (30.0)	0.24 #

# Fisher's exact test, * Mann-Whitney U test, ICM: Iodine contrast media

CO_2_: Carbon dioxide, BMI: Body mass index.

**Table 2:** Surgical outcomes.

	GROUPS		
		ICM	CO_2_	*p*-value
**Palpable femoral pulse pos n (%)**	8 (88.9%)	8 (80%)	>0.99 #
**Median ABI variation**	0.40	0.16	0.044 *

# Fisher exact test, * Mann-Whitney U test, ICM: Iodine contrast media

CO_2_: Carbon dioxide, Pos: Immediate post-operative period, ABI: Ankle brachial index.

**Table 3:** Comparison of pre- and post-operative creatinine clearance and delta clearance between the groups.

	ICM GROUP n=10	CO_2_ GROUP n=11	*p*-value#
**Median creatinine clearance pre (IIQ)**	90.20 (64.04-112.65)	79.40 (74.29-108.74)	0.94
**Median creatinine clearance pos (IIQ)**	93.05 (75.55-99.16)	88.40 (69.93-131.88)	0.62
**Median delta clearance**	0 (-16.9-10.28)	2.01 (-7.65-13.54)	0.44

# Mann-Whitney U test, IIQ: Interquartile interval, ICM: Iodine contrast media, CO_2_: Carbon dioxide, Pre: Before procedure, Pos: Post-operative period, Delta clearance: Creatinine clearance after the procedure minus clearance before the procedure.

**Table 4:** Endovascular material costs and contrast costs between the CO_2_ and iodine contrast media groups.

	GROUPS
	CO2	ICM	*p*-value #
**Endovascular material cost (IIQ)**	1527.69 (1527.6 9-1789.23)	2302.69 (1540.19-2984.07)	0.168
**Contrast cost (IIQ)**	10.12 (10.12-10.12)	25.0 (25.0-25.0)	<0.001

# Mann-Whitney U test, IIQ: Interquartile interval, ICM: Iodine contrast media, CO2: Carbon dioxide, values in American dollars.

**Table 5:** Evaluation of CO_2_ angiography by observers.

	Ilio-femoral angiography		
	Good	Fair	Poor
**Observer 1**	7	2	2
**Observer 2**	5	4	2
